# Supplementary material for: Use of screens, books and adults’ interactions on toddler’s language and motor skills: A cross-cultural study among 19 Latin American countries from different SES
Source: PLoS One. 2025 Feb 5;20(2):e0314569. doi: 10.1371/journal.pone.0314569 (PMC11798450; doi:10.1371/journal.pone.0314569)
Supplement: S1 Table — (DOCX) [file pone.0314569.s002.docx]

Supplementary Table 1

*Country-level sample data*

| Country | N | HDI | GII |
| --- | --- | --- | --- |
| Argentino/a | 166 | 0.842 | 0.287 |
| Boliviano/a | 68 | 0.692 | 0.418 |
| Brasileiro/a | 99 | 0.754 | 0.39 |
| Chileno/a | 100 | 0.855 | 0.187 |
| Colombiano/a | 91 | 0.752 | 0.424 |
| Costarricense | 92 | 0.809 | 0.256 |
| Cubano/a | 32 | 0.764 | 0.303 |
| Dominicano/a | 40 | 0.767 | 0.429 |
| Ecuatoriano/a | 87 | 0.74 | 0.362 |
| Guatemalteco/ca | 43 | 0.627 | 0.481 |
| Hondureño/ña | 32 | 0.621 | 0.431 |
| Mexicano/a | 91 | 0.758 | 0.309 |
| Nicaragüense | 87 | 0.667 | 0.424 |
| Panameño/a | 101 | 0.805 | 0.392 |
| Paraguayo/a | 86 | 0.717 | 0.445 |
| Peruano/a | 519 | 0.762 | 0.38 |
| Uruguayo/a | 10 | 0.809 | 0.235 |
| Venezolano/a | 50 | 0.691 | 0.492 |
