# Supplementary material for: Use of screens, books and adults’ interactions on toddler’s language and motor skills: A cross-cultural study among 19 Latin American countries from different SES
Source: PLoS One. 2025 Feb 5;20(2):e0314569. doi: 10.1371/journal.pone.0314569 (PMC11798450; doi:10.1371/journal.pone.0314569)
Supplement: Attachment — Submitted filename: Response to Reviewers.docx [file pone.0314569.s004.docx]

When submitting your revision, we need you to address these additional requirements.

1. Please ensure that your manuscript meets PLOS ONE's style requirements, including those for file naming. The PLOS ONE style templates can be found at

https://journals.plos.org/plosone/s/file?id=wjVg/PLOSOne_formatting_sample_main_body.pdf and

[https://journals.plos.org/plosone/s/file?id=ba62/PLOSOne_formatting_sample_title_authors_affiliations.pdf](https://c05y1x9s.r.us-east-2.awstrack.me/L0/https:%2F%2Fjournals.plos.org%2Fplosone%2Fs%2Ffile%3Fid=ba62%2FPLOSOne_formatting_sample_title_authors_affiliations.pdf/1/010f01920f975fac-ca9811b4-9b5e-490e-9a45-e9088543707f-000000/ugJMZ0c6K6mUVeW4WF1QfpUSpHY=177)

**The manuscript meets PLOS ONE's style requirements.**

2. Please include a complete copy of PLOS’ questionnaire on inclusivity in global research in your revised manuscript. Our policy for research in this area aims to improve transparency in the reporting of research performed outside of researchers’ own country or community. The policy applies to researchers who have travelled to a different country to conduct research, research with Indigenous populations or their lands, and research on cultural artefacts. The questionnaire can also be requested at the journal’s discretion for any other submissions, even if these conditions are not met.  Please find more information on the policy and a link to download a blank copy of the questionnaire here: https://journals.plos.org/plosone/s/best-practices-in-research-reporting. Please upload a completed version of your questionnaire as Supporting Information when you resubmit your manuscript.

**A complete copy of PLOS’ questionnaire on inclusivity in global research was included.**

3. We note that your Data Availability Statement is currently as follows: [All relevant data are within the manuscript and its Supporting Information files.] Please confirm at this time whether or not your submission contains all raw data required to replicate the results of your study. Authors must share the “minimal data set” for their submission. PLOS defines the minimal data set to consist of the data required to replicate all study findings reported in the article, as well as related metadata and methods (https://journals.plos.org/plosone/s/data-availability#loc-minimal-data-set-definition). For example, authors should submit the following data: - The values behind the means, standard deviations and other measures reported; - The values used to build graphs; - The points extracted from images for analysis. Authors do not need to submit their entire data set if only a portion of the data was used in the reported study. If your submission does not contain these data, please either upload them as Supporting Information files or deposit them to a stable, public repository and provide us with the relevant URLs, DOIs, or accession numbers. For a list of recommended repositories, please see https://journals.plos.org/plosone/s/recommended-repositories. If there are ethical or legal restrictions on sharing a de-identified data set, please explain them in detail (e.g., data contain potentially sensitive information, data are owned by a third-party organization, etc.) and who has imposed them (e.g., an ethics committee). Please also provide contact information for a data access committee, ethics committee, or other institutional body to which data requests may be sent. If data are owned by a third party, please indicate how others may request data access.

**The complete database and the analysis made are available in the manuscript, with an OSF URL.**

4. Please include your full ethics statement in the ‘Methods’ section of your manuscript file. In your statement, please include the full name of the IRB or ethics committee who approved or waived your study, as well as whether or not you obtained informed written or verbal consent. If consent was waived for your study, please include this information in your statement as well.

**A full ethics statement was included in the procedure section.**

5. Please review your reference list to ensure that it is complete and correct. If you have cited papers that have been retracted, please include the rationale for doing so in the manuscript text, or remove these references and replace them with relevant current references. Any changes to the reference list should be mentioned in the rebuttal letter that accompanies your revised manuscript. If you need to cite a retracted article, indicate the article’s retracted status in the References list and also include a citation and full reference for the retraction notice.
**The reference list was reviewed. Thank you for the follow up and feedback.**

Reviewers' comments:

Reviewer's Responses to Questions

**Comments to the Author**

1. Is the manuscript technically sound, and do the data support the conclusions?

The manuscript must describe a technically sound piece of scientific research with data that supports the conclusions. Experiments must have been conducted rigorously, with appropriate controls, replication, and sample sizes. The conclusions must be drawn appropriately based on the data presented.

Reviewer #1: Yes

**Thank you for your positive feedback.**

2. Has the statistical analysis been performed appropriately and rigorously?

Reviewer #1: Yes

**Thank you for your positive feedback.**

3. Have the authors made all data underlying the findings in their manuscript fully available?

The [PLOS Data policy](http://c05y1x9s.r.us-east-2.awstrack.me/L0/http:%2F%2Fwww.plosone.org%2Fstatic%2Fpolicies.action%23sharing/1/010f01920f975fac-ca9811b4-9b5e-490e-9a45-e9088543707f-000000/nDWaNFNGPR3HHtMam4Clj0nmKeI=177) requires authors to make all data underlying the findings described in their manuscript fully available without restriction, with rare exception (please refer to the Data Availability Statement in the manuscript PDF file). The data should be provided as part of the manuscript or its supporting information, or deposited to a public repository. For example, in addition to summary statistics, the data points behind means, medians and variance measures should be available. If there are restrictions on publicly sharing data—e.g. participant privacy or use of data from a third party—those must be specified.

Reviewer #1: Yes

**Thank you for your positive feedback.**

4. Is the manuscript presented in an intelligible fashion and written in standard English?

PLOS ONE does not copyedit accepted manuscripts, so the language in submitted articles must be clear, correct, and unambiguous. Any typographical or grammatical errors should be corrected at revision, so please note any specific errors here.

Reviewer #1: Yes

**Thank you for your positive feedback.**

5. Review Comments to the Author

Please use the space provided to explain your answers to the questions above. You may also include additional comments for the author, including concerns about dual publication, research ethics, or publication ethics. (Please upload your review as an attachment if it exceeds 20,000 characters)

Reviewer #1: Greetings.
Overall the write up is standard. However, I would suggest the following adjustments for upgrading the readability. This is an impressive endeavor and I wish for such important findings to be made public.

Introduction:
Please reduce the number of sentences. The introduction is very long. For each point of the introduction, try to limit the sentence count to 10 or fewer. Although the information provided is relevant, it can be more concise.

**The introduction was reduced from 3016 to 2796 words.**

Methodology-Analytical Plan:
Please rewrite this part in sub-sections or paragraphs addressing each of the analyses conducted in detail. The analyses are presented in the results section; however, the details should be mentioned here. It would be best to specify which statistical analysis was conducted for what reason, the variables included, and the rationale for their inclusion. Almost all information is already in this manuscript, but listing the analyses and their background in the Analytical Plan section of the Methodology is more appropriate. In addition to "In addition, we conducted multiple group comparisons to assess the existence of differences in the
use of screens according to the gender, age, and nationality of the children." please include which tests were done too.

**Different paragraphs with each statistical test analysis made in the manuscript were included in detail in the Analytical Plan. The new different paragraphs follow the same order as the results sections to facilitate the comprehension of the article.**

Result:

Please reorganize the results section to follow a more systematic approach. Remove the analysis details from this section and include them in the Analytical Plan. Ensure that tables or figures are provided for all respective results. Currently, it is difficult to discern which analysis yielded which result; this can be improved by using cross-referencing and organizing the results sequentially. For example, in the results section, one paragraph mentions:

"Partial correlations aimed to examine links between screen use (e.g., time, sharing, content)
and language and motor development in sample when controlling for child characteristics and
sociodemographic variables that relate to development. For child characteristics in the present
study, we found that age related to better language and earlier milestones while female children
get more scores in lexical density (U= 405, p= .003, r= .079) and language milestones (U= 399,
p< .001, r= .093). For sociodemographic variables we found that higher caregiver education was
related to better language and earlier milestones and access to daycare was related to more scores
in parents' reports of lexical density (X²= 73.39, p< .001, ε²= .039), use of sentences (X²= 46.95,
p< .001, ε²= .025) and earlier acquisition of language development milestones (X²= 35.56, p< .001,
ε²= .019). Thus age, gender, caregiver education, and daycare access were included as control
variables in Spearman parietal correlations to determine whether links between screen use,
language, and motor development existed when controlling for these variables. Partial correlations
for all variables are reported in Table 2."

There is no mention of where these results were calculated, nor are there any tables or figures referencing them. " For child characteristics in the present
study, we found that age related to better language and earlier milestones while female children
get more scores in lexical density (U= 405, p= .003, r= .079) and language milestones (U= 399,
p< .001, r= .093). " and "For sociodemographic variables we found that higher caregiver education was
related to better language and earlier milestones and access to daycare was related to more scores
in parents' reports of lexical density (X²= 73.39, p< .001, ε²= .039), use of sentences (X²= 46.95,
p< .001, ε²= .025) and earlier acquisition of language development milestones (X²= 35.56, p< .001,
ε²= .019). ".


In this paragraph, multiple test results are written together. The rationale for conducting these tests should be outlined in the Analytic Plan section, along with an explanation of why these results are relevant enough to be included alongside the partial correlation findings. For clarity, it would be beneficial to present each test result in separate paragraphs, enhancing comprehension for non-statistically inclined readers. Simplifying the presentation of these critical findings is necessary to ensure they are accessible and understandable to a broader audience. Please rewrite or reorganize the result section to improve the readability.

**The results section was reorganized to follow a more systematic approach. The analysis details previously included in the results section were moved to the Analytic Plan. Additionally, each test result is now presented in separate paragraphs, with distinct subtitles to better organize the information. Finally, we simplified the content, highlighting the most important findings to enhance the article's readability and comprehension.**

Discussion and Conclusion: No changes are required.

**Thank you for your positive feedback.**

6. PLOS authors have the option to publish the peer review history of their article ([what does this mean?](https://c05y1x9s.r.us-east-2.awstrack.me/L0/https:%2F%2Fjournals.plos.org%2Fplosone%2Fs%2Feditorial-and-peer-review-process%23loc-peer-review-history/1/010f01920f975fac-ca9811b4-9b5e-490e-9a45-e9088543707f-000000/pRs8-7-ADeIQkvmQ9j43LFh_ulA=177)). If published, this will include your full peer review and any attached files.


If you choose “no”, your identity will remain anonymous but your review may still be made public.


**Do you want your identity to be public for this peer review?** For information about this choice, including consent withdrawal, please see our [Privacy Policy](https://c05y1x9s.r.us-east-2.awstrack.me/L0/https:%2F%2Fwww.plos.org%2Fprivacy-policy/1/010f01920f975fac-ca9811b4-9b5e-490e-9a45-e9088543707f-000000/M2W9RyvkcUlxUcHsjgT2cUab8Zs=177).

Reviewer #1: No
